# DDAH1 Protects against Acetaminophen-Induced Liver Hepatoxicity in Mice

**DOI:** 10.3390/antiox11050880

**Published:** 2022-04-29

**Authors:** Xiyue Shen, Saddam Muhammad Ishaq, Qiao’e Wang, Juntao Yuan, Junling Gao, Zhongbing Lu

**Affiliations:** 1 College of Life Sciences, University of Chinese Academy of Sciences, Beijing 100049, China; shenxiyue16@mails.ucas.ac.cn (X.S.); saddamdabai1@gmail.com (S.M.I.); yuanjuntao@ucas.ac.cn (J.Y.); gaojunling17@mails.ucas.ac.cn (J.G.); 2 Key Laboratory of Cosmetic, China National Light Industry, Beijing Technology and Business University, Beijing 100048, China; wangqe@th.btbu.edu.cn

**Keywords:** DDAH1, acetaminophen, oxidative stress, inflammation, liver injury

## Abstract

In many developed countries, acetaminophen (APAP) overdose-induced acute liver injury is a significant therapeutic problem. Dimethylarginine dimethylaminohydrolase 1 (DDAH1) is a critical enzyme for asymmetric dimethylarginine (ADMA) metabolism. Growing evidence suggests that liver dysfunction is associated with increased plasma ADMA levels and reduced hepatic DDAH1 activity/expression. The purpose of this study was to investigate the involvement of DDAH1 in APAP-mediated hepatotoxicity using *Ddah1*^-/-^ and DDAH1 transgenic mice. After APAP challenge, *Ddah1*^-/-^ mice developed more severe liver injury than wild type (WT) mice, which was associated with a greater induction of fibrosis, oxidative stress, inflammation, cell apoptosis and phosphorylation of JNK. In contrast, overexpression of DDAH1 attenuated APAP-induced liver injury. RNA-seq analysis showed that DDAH1 affects xenobiotic metabolism and glutathione metabolism pathways in APAP-treated livers. Furthermore, we found that DDAH1 knockdown aggravated APAP-induced cell death, oxidative stress, phosphorylation of JNK and p65, upregulation of CYP2E1 and downregulation of GSTA1 in HepG2 cells. Collectively, our data suggested that DDAH1 has a marked protective effect against APAP-induced liver oxidative stress, inflammation and injury. Strategies to increase hepatic DDAH1 expression/activity may be novel approaches for drug-induced acute liver injury therapy.

## 1. Introduction

Acetaminophen (APAP), also known as paracetamol or N-acetyl-P-aminophenol, is a safe and efficient analgesic and antipyretic medicine when taken as directed. However, patients can consciously or unconsciously overdose APAP due to its abundance, which results in liver injury and acute liver failure [1]. Indeed, APAP overdose is the leading cause of acute liver failure in the United States and many other Western countries. 

After overdose, part of APAP is metabolized by the cytochrome P450 enzymes into a very toxic metabolite termed N-acetyl-P-benzo quinone imine (NAPQI) [2]. The excessive NAPQI formed during APAP overdose depletes intracellular glutathione (GSH) and covalently binds to sulfhydryl groups of multiple proteins, especially mitochondrial proteins, resulting in mitochondrial oxidative stress and dysfunction. Although extensive research has been performed on the mechanisms of drug-induced liver injury (DILI) in order to create novel therapeutic strategies, existing therapy options for APAP overdose are immensely limited [3]. In addition, DILI is a completely unpredictable disease. As a result, rational and effective approaches to the prevention and treatment of APAP-induced liver toxicity are urgently needed.

As an endogenous inhibitor of nitric oxide (NO) synthases (NOS), asymmetric dimethylarginine (ADMA) can not only decrease NO production but also increase the production of NOS-derived reactive oxygen species (ROS) under some pathological conditions [4]. Numerous studies have demonstrated that increased circulating ADMA levels were found in individuals suffering APAP-induced acute liver injury (ALI) [5], nonalcoholic fatty liver disease (NAFLD) [6], cirrhosis [7] and alcoholic hepatitis [8]. ADMA is mainly degraded by dimethylarginine dimethylaminohydrolase 1 (DDAH1), an enzyme that is extensively manifested in mice [9]. Impaired DDAH1 activity and lowered DDAH1 expression in the liver are likely to be the mechanisms by which liver dysfunction leads in elevated ADMA levels [10,11]. We previously demonstrated that DDAH1 has profound effects on cellular redox state [12,13] and *Ddah1* deletion exacerbated high-fat diet (HFD)-induced hepatic steatosis and oxidative stress in mice [14]. However, the role of DDAH1 in APAP-induced ALI remains unclear. 

In this study, genetic background matched *Ddah1*^-/-^, DDAH1 transgenic (DDAH1-TG), and wild-type (WT) mice were used to explore the effect of DDAH1 on APAP-induced liver dysfunction and hepatic oxidative stress.

## 2. Materials and Methods

### 2.1. Reagents and Antibodies

GSH assay and TUNEL staining kits were purchased from the Beyotime Institute of Biotechnology (#S0053, #C1090 and #P0012S, Shanghai, China). Alanine aminotransferase (ALT) and aspartate aminotransferase (AST) kits were purchased from Nanjing Jiancheng Bioengineering Institute (#C009–2 and #C010–2, Nanjing, Jiangsu, China). Elisa kits for ADMA and 4-hydroxynonenal (4-HNE) were purchased from Bio-Techne Co., Ltd. (#NBP2–66728, Minneapolis, MN, USA) and Donggeboye Biological Technology Co., LTD. (#DG30947M, Beijing, China), respectively. The Masson’s trichrome staining kit was obtained from Solarbio Science & Technology Co. LTD (#G1340, Beijing, China). Antibodies against DDAH1, cytochrome P450 2E1 (CYP2E1), glutathione S-transferase A1 (GSTA1) and β-actin were purchased from Signalway Antibody LLC (#37368, #48247, #22536, #21800, Greenbelt, MD, USA). P65, phospho-65, c-jun N-terminal kinase (JNK) and phospho-JNK antibodies were from Cell Signaling Technology (#8242, #3033, #9252, #9251, Danvers, MA, USA). APAP and dihydroethidium (DHE) were purchased from MedChemExpress LLC (#HY-66005, Monmouth Junction, NJ, USA) and Sigma Chemical Co. (#D7008, St. Louis, MO, USA), respectively. All other compounds and chemicals were of analytical purity.

### 2.2. Animals 

The original *Ddah1*^-/-^ mice [9] were crossed with C57BL/6J mice for more than 10 generations. DDAH1 transgenic mice (DDAH1-TG, C57BL/6J background) were generated by Cyagen Biosciences Inc (Jiangsu, China) using an expression vector containing an EF1A promoter, 3xFLAG/human DDAH1 [ORF003160] and RNA processing signals from SV40. Male, 8-week-old C57BL/6J, *Ddah1*^-/-^ and DDAH1-TG mice were divided into control and APAP-treated groups at random (8 mice/group). The mean bodyweight was about 21–23 g. Mice were kept in a temperature-controlled room under a 12:12 h light-dark cycle. The animals had free access to drinking water and were fed a standard chow diet. The animals were fasted overnight before the studies, with water available ad libitum. APAP was dissolved in 0.9% saline (30 mg/mL) and intraperitoneally administered at a dose of 300 mg/kg. Mice were euthanized using spinal cord dislocation method 48 h after APAP administration, and then blood was collected from orbital sinus and liver tissue was collected. During the whole experimental period, mice were treated in line with the principles of laboratory animal care (NIH publication no. 85–23, revised 1985).

### 2.3. Histological Analyses

The mouse livers were collected after perfusion and preserved in 4% formaldehyde solution. Subsequently, they were dehydrated with increasing concentrations of alcohol (50–100%) and paraffin-embedded and sliced in semi-serial at a 5 μm thickness. Liver paraffin sections were stained with hematoxylin and eosin (H&E), Masson’s trichrome stain kit and TUNEL stain kit, respectively. To assess hepatic superoxide levels, frozen liver sections (4 μm) were stained with DHE.

### 2.4. RNA Extraction and Sequencing 

Total RNA was extracted from the livers using the TRIzol reagent. To assess RNA quality, the Agilent 2100 bioanalyzer (Thermo Fisher Scientific, Waltham, MA, USA) was utilized, and samples with an RNA integrity number greater than 8 were used in further investigations. RNA was further purified by digesting double-stranded and single-stranded DNA with DNase I and removing rRNA with the Ribo-Zero method (human, mouse, plants) (Illumina, San Diego, CA, USA). A BGISEQ500 platform was used for library construction and RNA sequencing (BGI-Shenzhen, Shenzhen, China). The details for raw data cleaning, mapping, gene expression level assessment, differentially expressed gene (DEG) screening and Kyoto Encyclopedia of Genes and Genomes (KEGG) enrichment analysis were described in our previous reports [15,16]. 

### 2.5. Cell Culture

HepG2 cells were taken from the Institute of Biochemistry and Cell Biology’s Cell Bank and cultured at 37 °C with 5% CO_2_ in DMEM supplemented with 25 mM glucose, 10% FBS, and 1% penicillin and streptomycin. As previously reported [13], cell viability and intracellular superoxide levels were determined. The stable DDAH1 knockdown cell line was generated using the PLKO-shDDAH1 lentivirus (target sequence: GGGCCTAACCTGATCGCAATT). 

### 2.6. Real Time qPCR and Western Blot

The quantitative real-time polymerase chain reaction (qPCR) assay was performed with the SYBR^®^ Premix Ex Taq™ II Kit (#RR820A). 18S ribosomal RNA was used to normalize relative expression of target genes. The primer sequences used are listed in Table 1. The PCR product lengths are as follows: 18S (127 bp), TNFα (103 bp), TGFβ (94 bp), collagen I (110 bp) and collagen III (98 bp).

Protein extractions were performed with a lysis buffer comprising 150mM NaCl, 100 μg/mL phenylmethylsulfonyl fluoride, 50 mM Tris-Cl, 1% Triton X-100 and protease and phosphatase inhibitor cocktail (#04693124001, #4906837001, Roche, Basel, Switzerland). After centrifugation at 12,000× *g* and 4 °C for 20 min, the supernatant was employed for Western blot analysis. First, equal amounts of protein (10–40 μg) were loaded and separated by SDS-PAGE gels. Then, the gels were transferred to polyvinylidene fluoride membranes. After that, the membranes were blocked for 1 h at room temperature with 5% non-fat milk dissolved in TBS-T buffer (50 mmol/L Tris-HCl,150 mmol/L NaCl and 0.1% Tween 20). Next, the membranes were incubated with the indicated primary antibodies (1:1000) overnight with constant agitation at 4 °C. After thoroughly washing, the membranes were incubated with the corresponding horseradish peroxidase-labeled secondary antibodies (1:5000) for 1 h at room temperature with constant agitation, then washed and reacted with the chemiluminescent substrate. The blots were visualized using ChemiDoc™ XRS+ Gel Imaging System (Bio-Rad Laboratories, Inc., Hercules, CA, USA).

### 2.7. Statistical Analysis

All data were analyzed with GraphPad Prism 8 (GraphPad Software Inc., San Diego, CA, USA) and expressed as mean ± SD. To make numerous comparisons between groups, an unpaired 2-tailed t test or one-way ANOVA followed by Fisher’s least significant difference test or the Kruskal–Wallis nonparametric test followed by Dunn’s test was utilized. *p* < 0.05 was defined as statistically significant.

## 3. Results

### 3.1. DDAH1 Protects against APAP-Induced Liver Dysfunction and Fibrosis

**Figure 1:**
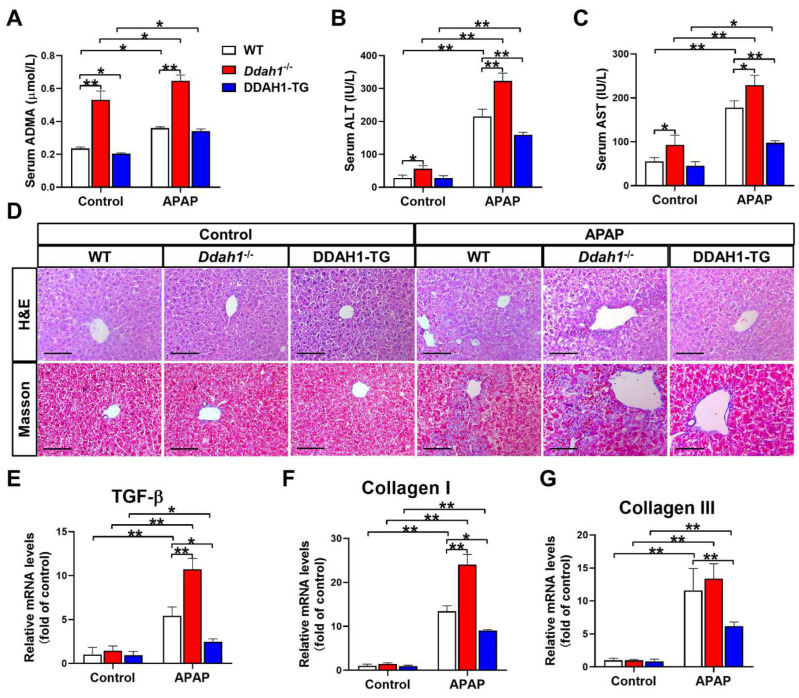
DDAH1 alleviates liver dysfunction and fibrosis in acetaminophen-treated mice. Male genetic background-matched wild-type (WT), *Ddah1*^-/-^ and DDAH1 transgenic (DDAH1-TG) mice were treated with acetaminophen (APAP) for 48 h. Serum ADMA (**A**), alanine transaminase (ALT) (**B**), and aspartate transaminase (AST) (**C**) levels were measured. (**D**) Representative liver sections were stained with hematoxylin and eosin (H&E) and Masson. Scale bar = 100 μm. (**E**–**G**) The mRNA levels of fibrotic genes were measured by real-time qPCR. *n* = 5, values are expressed as means ± SD; * indicates *p* < 0.05; ** indicates *p* < 0.01.

As a critical enzyme for ADMA degradation, deletion of *Ddah1* increased (0.531 ± 0.054 vs. 0.236 ± 0.009) whereas overexpression of DDAH1 decreased (0.205 ± 0.004 vs. 0.236 ± 0.009) serum ADMA levels in control mice. APAP treatment resulted in significant increases in serum ADMA levels. However, *Ddah1*^-/-^ mice still exhibited higher serum ADMA levels than WT mice (0.647 ± 0.036 vs. 0.359 ± 0.008) (Figure 1A). *Ddah1* deletion also significantly increased serum ALT and AST levels in control mice. After APAP treatment, serum ALT and AST levels were significantly increased in WT (ALT: 215.4 ± 21.7 vs. 28.5 ± 8.2; AST: 118.0 ± 15.4 vs. 55.2 ± 9.0), *Ddah1*^-/-^ (ALT: 322.7 ± 24.1 vs. 56.2 ± 9.8; AST: 229.2 ± 22.2 vs. 92.9 ± 22.0) and DDAH1-TG mice (ALT: 159.4 ± 7.6 vs. 28.1 ± 7.2; AST: 97.7 ± 4.5 vs. 45.2 ± 9.0). However, the APAP-induced increases in serum ALT and AST levels were exacerbated by Ddah1 deletion, whereas they were attenuated by DDAH1 overexpression (Figure 1B,C).

H&E staining of liver sections revealed that APAP-induced pathological changes, including centrilobular hepatic necrosis, inflammatory cell infiltration and ballooning degeneration, were significantly aggravated in the *Ddah1*^-/-^ mice. APAP treatment also caused more liver fibrosis in the *Ddah1*^-/-^ mice than in the WT mice. Compared with the livers of WT mice, livers from DDAH1-TG mice developed less severe injury and fibrosis in response to APAP (Figure 1D). APAP significantly increased TGF-β, collagen I and III mRNA levels in the livers of mice of the three genotypes. However, the upregulation of TGF-β and collagen I was exacerbated in the livers of *Ddah1*^-/-^ mice and those upregulations were alleviated in the livers of DDAH1-TG mice (Figure 1E–G). 

### 3.2. DDAH1 Attenuates APAP-Induced Hepatic Oxidative Stress and Apoptosis

**Figure 2:**
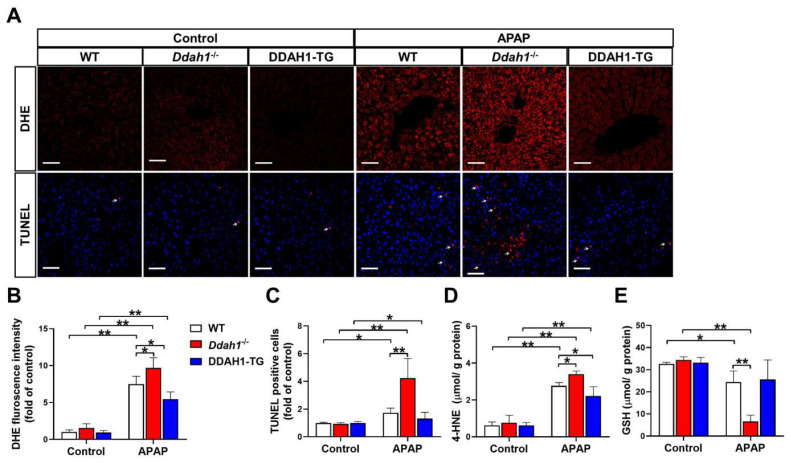
DDAH1 alleviates oxidative stress and apoptosis in APAP-treated mice. (**A**) Liver sections from control and APAP-exposed mice were stained with dihydroethidium (DHE) and a TUNEL assay kit (red) and DAPI (blue) (scale bar = 50 μm). The relative fluorescence intensities of DHE (**B**) and the TUNEL-positive cells (**C**) were quantified. After APAP treatment, 4-HNE (**D**) and GSH (**E**) levels in the liver were measured. *n* = 5, values are expressed as means ± SD; * indicates *p* < 0.05; ** indicates *p* < 0.01.

It has been reported that the hepatotoxicity of APAP is closely related with oxidative stress and apoptosis [17]. To assess the effect of APAP treatment on hepatic ROS generation and apoptosis, liver sections were stained with DHE and TUNEL, respectively. In the livers of control mice, the fluorescence intensity of DHE was very weak, and there were little TUNEL-positive cells (indicated by the arrow). APAP treatment significantly increased DHE fluorescence intensity and TUNEL-positive cells in liver sections from WT, *Ddah1*^-/-^ and DDAH1-TG mice. However, liver sections from *Ddah1*^-/-^ exhibited higher DHE fluorescence intensity and more TUNEL-positive cells than those of WT mice, indicating that APAP caused higher superoxide levels and more apoptotic cells in the livers of *Ddah1*^-/-^ mice than in WT mice. In addition, DDAH1 overexpression did not affect the number of apoptotic cells but decreased hepatic superoxide levels in APAP-challenged mice (Figure 2A–C). To further confirm that DDAH1 affects APAP-induced hepatic oxidative stress, the levels of 4-HNE and GSH were also measured. APAP challenge resulted in significant increases in 4-HNE levels and decreases in GSH levels, and these changes were significantly greater in the livers of *Ddah1*^-/-^ mice. DDAH1 overexpression decreased 4-HNE levels but had no obvious effect on GSH levels (Figure 2D,E). 

### 3.3. DDAH1 Inhibits APAP-Induced Inflammation

**Figure 3:**
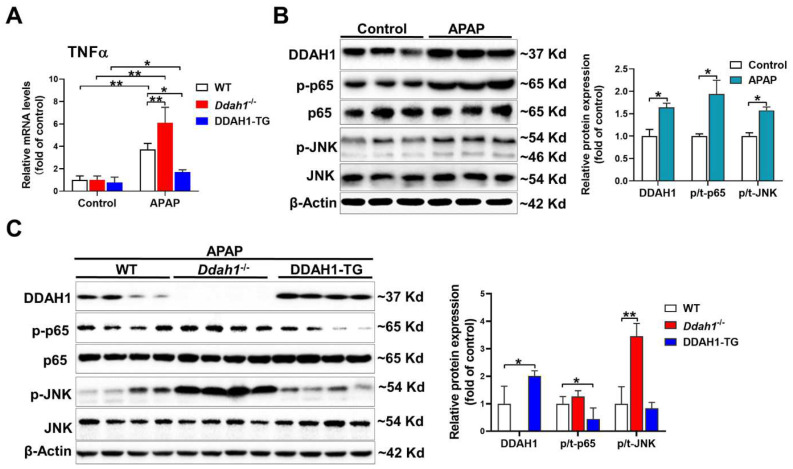
DDAH1 attenuated hepatic inflammatory response in APAP-treated mice. (**A**) The mRNA levels of TNFα were measured by real-time qPCR. (**B**,**C**) Liver lysates were examined by Western blot. *n* = 3–5, values are expressed as means ± SD; * indicates *p* < 0.05; ** indicates *p* < 0.01.

As inflammation is also involved in the hepatotoxicity of APAP, we measured the mRNA levels of TNFα. APAP significantly increased TNFα mRNA levels in the livers of mice of three genotypes. However, the upregulation of hepatic TNFα was greater in the *Ddah1*^-/-^ mice and was alleviated in the DDAH1-TG mice (Figure 3A). After APAP challenge, hepatic DDAH1 expression was increased about ~60%. DDAH1 was undetected in the livers of *Ddah1*^-/-^ mice, and DDAH1-TG mice exhibited higher hepatic DDAH1 levels than WT mice (Figure 3B). APAP treatment also increased the expression of the phosphorylation of NFκB p65 (Ser536) and JNK (Thr183/Tyr185) (Figure 3A). Interestingly, *Ddah1* deletion exacerbated the increases in p-JNK levels but had no obvious effect onp-p65 levels. DDAH1 overexpression attenuated the APAP-induced upregulation of p-p65 but did not affect p-JNK levels (Figure 3B).

### 3.4. DDAH1 Affects the Gene Expression Profile in APAP-Challenged Mouse Livers

**Figure 4:**
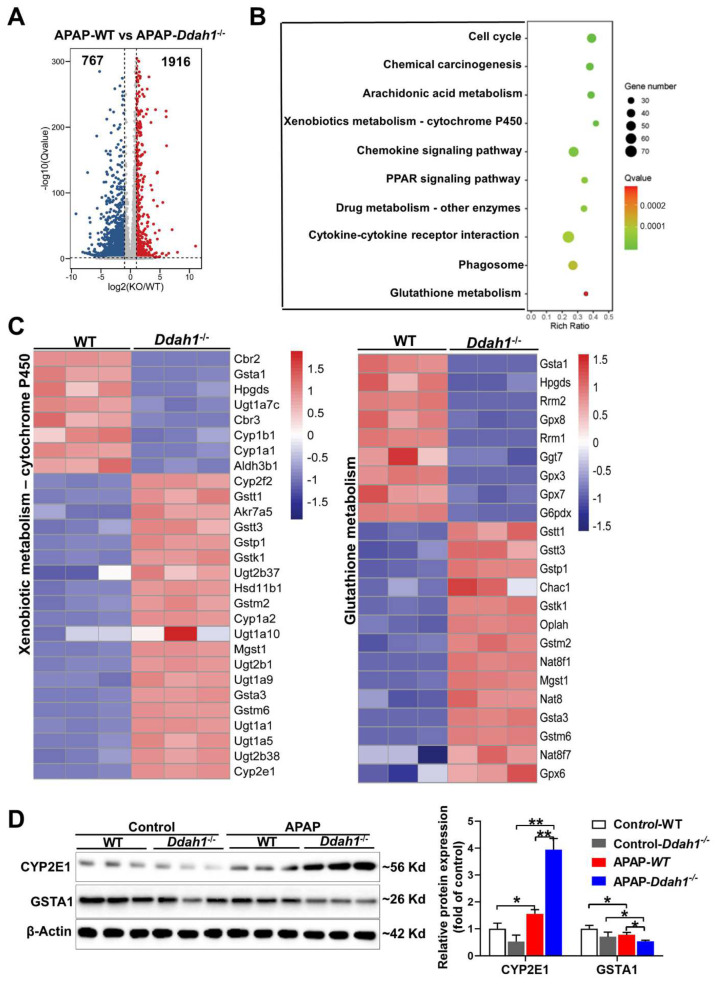
DDAH1 affects the gene expression profile in APAP-treated livers. (**A**) The volcano plot shows the fold changes of differentially expressed genes (DEGs) in the APAP-WT group vs. APAP-*Ddah1*^-/-^ group. (**B**) The selected significantly enriched KEGG pathways were listed as advanced bubble charts. (**C**) The gene expression profiles of the DEGs involved in xenobiotic metabolism and glutathione metabolism pathways, as determined by KEGG pathway analysis, are shown in the heat maps. (**D**) Liver lysates from control and APAP-treated WT and *Ddah1*^-/-^ mice were examined by Western blot. *n* = 3; values are expressed as means ± SD; * indicates *p* < 0.05; ** indicates *p* < 0.01.

To further reveal the molecular mechanism by which DDAH1 protects against APAP-induced liver injury, the changes in the whole-genome expression profile of APAP-challenged livers were analyzed by RNA sequencing technique. We identified 1916 up-regulated and 767 down-regulated DEGs in the comparison of APAP-treated WT and *Ddah1*^-/-^ livers. The fold change of these identified DEGs was visualized by a volcano plot (Figure 4A). We also performed KEGG pathway enrichment analysis and found that the DEGs were mainly significantly enriched in metabolic and inflammatory pathways, including arachidonic acid metabolism, xenobiotic metabolism, glutathione metabolism, chemokine signaling pathway, and cytokine–cytokine receptor interaction pathways (Figure 4B). The expression of the DEGs involved in xenobiotic metabolism-cytochrome P450 and glutathione metabolism pathways was visualized in heatmaps (Figure 4C). Some detoxification or antioxidant related genes, including *Gsta1*, *Aldh3b1* and *GPX3/8/7*, were downregulated in *Ddah1*^-/-^ livers. GSTA1 is the main glutathione S-transferase in human liver and is a sensitive and accurate indicator of liver injury [18]. Western blot results showed that APAP increased CYP2E1 expression and decreased GSTA1 expression. The APAP-induced upregulation of CYP2E1 and downregulation of GSTA1 were aggravated in the livers of *Ddah1*^-/-^ mice (Figure 4D).

### 3.5. Knockdown of DDAH1 Promotes APAP-Induced Cell Death and Oxidative Stress in HepG2 Cells

**Figure 5:**
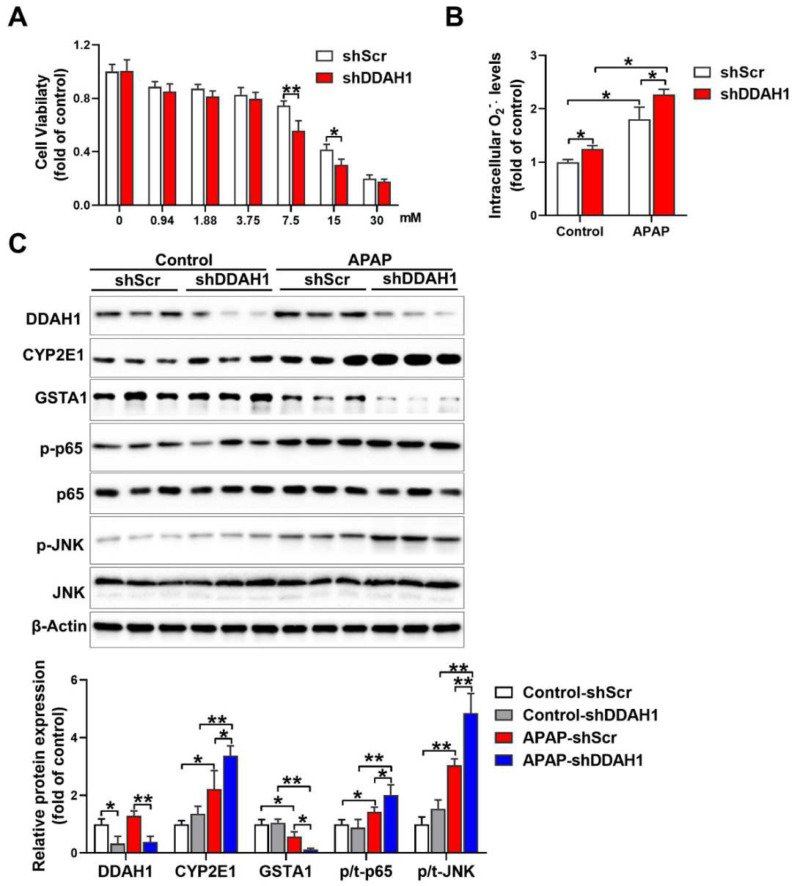
Effect of DDAH1 knockdown on APAP-induced cell death, oxidative stress and inflammatory pathways. (**A**) HepG2 cells stably transfected with shRNA lentiviral vectors targeting DDAH1 (shDDAH1) or a scrambled sequence (shScr) were treated with different concentrations of APAP for 24 h, and then cell viability was measured. (**B**) After incubation with 15 mM APAP for 24 h, the intracellular superoxide levels were determined. (**C**) Control and DDAH1-depleted cells were treated with 15 mM APAP for 24 h, and cell lysates were examined by Western blot. (**A**), *n* = 8; (**B**), *n* = 5; (**C**), *n* = 3; values are expressed as means ± SD; * indicates *p* < 0.05; ** indicates *p* < 0.01.

To confirm the protective role of DDAH1 in the hepatotoxicity of APAP, we stably transfected HepG2 cells with DDAH1-specific shRNA lentiviral vector (shDDAH1) or an shRNA lentiviral vector targeting a scrambled sequence (shScr). APAP treatment caused more reduction in cell viability in DDAH1-depleted cells than in control cells at the concentrations of 7.5 mM and 15 mM (Figure 5A). Under basal conditions, DDAH1 knockdown increased intracellular superoxide levels. After APAP challenge (15 mM, 24 h), there were significant increases in intracellular superoxide levels. However, compared to control cells, DDAH1-depleted cells exhibited higher levels of intracellular superoxide (Figure 5B). Western blot results showed that shDDAH1 transfection decreased DDAH1 expression by ~60%. In control cells, DDAH1 knockdown had no obvious effect on the expression of CYP2E1, GSTA1, p-p65 and p-JNK. After APAP treatment, DDAH1-depleted cells exhibited significantly higher levels of CYP2E1, p-p65 and p-JNK, as well as lower levels of GSTA1 (Figure 5C), suggesting that DDHA1 knockdown exacerbated APAP-induced inflammation and oxidative stress.

## 4. Discussion

Epidemiological studies have demonstrated that patients with liver diseases exhibit increased plasma ADMA levels. As a critical enzyme for ADMA degradation, DDAH1 is highly expressed in the liver and might play an important role in the pathophysiology of multiple liver diseases. For example, DDAH1 expression is reduced in the livers of bile duct-ligated (BDL) cirrhotic rats, and DDAH1 expression increased by TNF blockade or hydrodynamic injection with DDAH1-expressing plasmids significantly reduced ADMA content and portal pressure [19,20]. We previously showed that deletion of *Ddah1* exacerbated hepatic steatosis and insulin resistance in obese mice [14]. Some synthetic farnesoid X receptor (FXR) agonists, including GW4064 [21], obeticholic acid [19] and INT-747 [22], could increase hepatic DDAH1 expression and attenuate the development of NAFLD. In the present study, we demonstrated that DDAH1 was upregulated in the livers of APAP-treated mice and overexpression attenuated, whereas *Ddah1* deficiency exacerbated, APAP-induced liver dysfunction, fibrosis and cell apoptosis. We also showed that DDAH1 depletion aggravated APAP-induced cell death in HepG2 cells. These results suggested that DDAH1 exerts a protective role in APAP-induced liver injury. 

Excessive ROS generation from NAPQI formation during APAP overdose has been regarded as the predominant mechanism in APAP-induced hepatotoxicity [23]. The present study showed that DDAH1 overexpression decreased, whereas *Ddah1* deficiency increased, superoxide and 4-HNE levels in the livers of APAP-treated mice, suggesting that DDAH1 may exert hepatic protective effects through repressing oxidative stress. Since ADMA accumulation may cause oxidative stress via promoting NOS uncoupling or upregulating the renin–angiotensin system, it is possible that the impact of DDAH1 on hepatic redox state is associated with ADMA degradation. It is well established that NAPQI is mainly generated by the cytochrome P450 enzymes and detoxified by GSH and detoxification enzymes [2]. The present study showed that DDAH1 profoundly affects xenobiotic metabolism and glutathione metabolism pathways. Specifically, *Ddah1* deficiency increased CYP2E1 expression and decreased GSTA1 protein expression and GSH levels in APAP-treated livers. Thus, it is likely that DDAH1 may also ameliorate APAP-induced oxidative stress via inhibiting the formation of toxic metabolites and regulating the cellular antioxidant system. The regulating effect of DDAH1 on antioxidant enzymes has been also found in cell models [13,24], fatty livers [14] and aged and diabetic kidneys [12].

It has been reported that toxic APAP exposure is accompanied by an inflammatory response that mediates the second phase of liver injury [25,26]. NFκB and JNK, two key regulators of hepatic inflammation, are activated in APAP-treated livers, and administration of the NFκB inhibitor BAY 11-7082 or JNK inhibitor SP600125 protected against APAP-mediated liver injury [27,28]. We previously showed that *Ddah1* deficiency exacerbated inflammatory response and activation of NFκB and JNK in fatty livers [14]. In mouse embryonic fibroblasts, deletion of *Ddah1* directly activates NFκB [13]. Here, we also found that DDAH1 attenuated APAP-induced upregulation of TNFα and phosphorylation of JNK and p65 in livers and cells, suggesting that DDAH1 also protects against APAP-induced liver injury, at least partially, by suppressing inflammation. Since ADMA could activate JNK/NFκB in multiple cell lines [29,30,31], it is possible that DDAH1 represses JNK/NFκB activity via degrading ADMA. In addition, DDAH1 may also inhibit JNK activity via regulating GSTA1 expression in APAP-treated livers, which can interact with JNK in unstressed cells and suppress oxidative stress-mediated JNK activation [32,33].

## 5. Conclusions

In summary, our study demonstrates that DDAH1 protects against APAP-induced liver injury by degrading ADMA, regulating glutathione metabolism and attenuating JNK and NFκB activation, thereby decreasing oxidative stress and inflammation. These results suggest that DDAH1 is a potential therapy target for drug-induced acute liver injury. 

## Figures and Tables

**Table 1:** The quantitative real-time PCR primer information.

Genes	Accession	Primers	Sequence (5’-3’)
18 s	NM_011296.3	Forward	5′-TTCTGGCCAACGGTCTAGACAAC-3′
		Reverse	5′-CCAGTGGTCTTGGTGTGCTGA-3′
TNFα	NM_013693	Forward	5′- AGGGTCTGGGCCATAGAACT-3′
		Reverse	5′- CCACCACGCTCTTCTGTCTAC -3′
TGFβ	NM_011577	Forward	5′-CAACCCAGGTCCTTCCTAAA -3′
		Reverse	5′-GGAGAGCCCTGGATACCAAC-3′
Collagen I	NM_007742	Forward	5′-TAGGCCATTGTGTATGCAGC-3′
		Reverse	5′-ACATGTTCAGCTTTGTGGACC-3′
Collagen III	NM_009930	Forward	5′-TAGGACTGACCAAGGTGGCT-3′
		Reverse	5′-GGAACCTGGTTTCTTCTCACC-3′

## Data Availability

The data presented in this study are available on reasonable request from the corresponding author. The sequencing data for clean reads generated by this study have been deposited in the NCBI Sequence Read Archive (SRA) database (accession number: PRJNA822177).
